# Phytochemicals and Antioxidant Capacity from *Nypa fruticans* Wurmb. Fruit

**DOI:** 10.1155/2013/154606

**Published:** 2013-04-21

**Authors:** Nagendra Prasad, Bao Yang, Kin Weng Kong, Hock Eng Khoo, Jian Sun, Azrina Azlan, Amin Ismail, Zulfiki Bin Romli

**Affiliations:** ^1^Chemical Engineering Discipline, School of Engineering, Monash University, Jalan Lagoon Selatan, 46150 Bandar Sunway, Selangor, Malaysia; ^2^Department of Nutrition and Dietetics, Faculty of Medicine and Health Sciences, Universiti Putra Malaysia, 43400 Serdang, Selangor, Malaysia; ^3^Key Laboratory of Plant Resources Conservation and Sustainable Utilization, South China Botanical Garden, Chinese Academy of Sciences, Guangzhou 510650, China; ^4^Department of Molecular Medicine, Faculty of Medicine, University of Malaya, 50603 Kuala Lumpur, Malaysia; ^5^Institute of Agro-food Science and Technology, Guangxi Academy of Agricultural Sciences, Nanning 530007, China; ^6^Laboratory of Halal Science Research, Halal Products Research Institute, Universiti Putra Malaysia, 43400 Serdang, Selangor, Malaysia; ^7^MUDA Agricultural Development Authority (MADA), Alor Setar 05990, Kedah, Malaysia

## Abstract

*Nypa fruticans* Wurmb. is one of the important underutilized fruit of Malaysia, which lacks scientific attention. Total phenolics, flavonoid content, and antioxidant capacities from endosperm extracts of *Nypa fruticans* (unripe and ripe fruits) were evaluated. Endosperm extract of unripe fruits (EEU) exhibited the highest phenolics (135.6 ± 4.5 mg GAE/g), flavonoid content (68.6 ± 3.1 RE/g), and antioxidant capacity. Free radical scavenging capacity of EEU as assessed by 2-2′-azino-bis (3-ethylbenz-thiazoline-6-sulfonic acid (ABTS) and 1,1-diphenyl-2-picryl hydrazyl (DPPH) radicals showed inhibitory activity of 78 ± 1.2% and 85 ± 2.6%, respectively. Beta carotene bleaching coefficient of EEU was higher (2550 ± 123), when compared to endosperm extract of ripe fruits (1729 ± 172). Additionally, EEU exhibited high antioxidant capacity by phosphomolybdenum method and ferric reducing antioxidant power values. Eight phenolic compounds from *Nypa fruticans* endosperm extracts were identified and quantified by ultra-high-performance liquid chromatography. Chlorogenic acid, protocatechuic acid, and kaempferol were the major phenolic compounds. Thus this fruit could be used as a potential source of natural antioxidant.

## 1. Introduction

Antioxidants from plant sources have been an increasing concern to consumers since synthetic antioxidants such as butylated hydroxy toluene (BHT) have restricted usage in foods, due to carcinogenic activity [1]. Epidemiological studies have shown that frequent consumption of fruits and vegetables high in natural antioxidants can lower the incidence of certain types of cancer, cardiovascular diseases, and diabetes [2]. These beneficial effects are related to bioactive compounds like phenolic acids, flavonoids, anthocyanins, and carotenoids possessing antioxidant activity [3, 4].

The years 2011–2020 are recognized as the “Decade on Biodiversity” by the United Nations to promote the importance and utilization of underutilized foods. Recently, research and development activities on antioxidants from underutilized fruits have become a great priority [5, 6]. They are notable by the fact that they are locally available but universally erratic, and much related information is also limited. Many of these fruits have a wide range of color for skin and pulp with health-promoting benefits. However, many of these fruits are still not familiar due to lack of publicity and promotional campaign [7].

**Figure 1:**
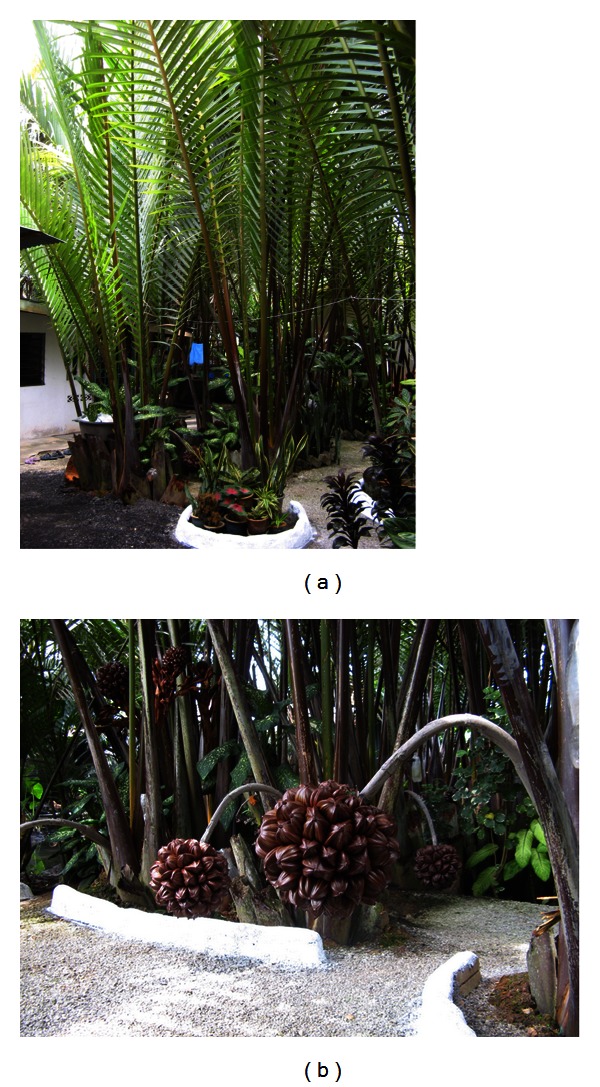
*Nypa fruticans* (a) and fruit bunch (b).

*Nypa fruticans* Wurmb. (NF) belongs to *Araceae* family and is considered as “underutilized” plant [8]. Other synonym of this plant includes *Cocos nypa* Lour., *Nypa fruticans* Thunb., and *Nypa palm*. It is a monoecious palm found growing in brackish water with upright stem (Figure 1(a)), trunkless with fruits found commonly emerging from the soil (Figure 1(b)). This palm is typically found in India, Malaysia, Indonesia, Philippines, and in some parts of Queensland, Australia [9]. Sap (obtained from the inflorescence stalk) is used as a drink by the indigenous peoples, while young fruits are eaten [10]. The sap is a good source of sugar and used for making sweets, vinegar, beverage, and alcohol production [8]. The fruit is also rich in carbohydrates, fibers, minerals, and vitamin A [11]. Traditionally, leaves, stem, and roots of NF are used to treat asthma, leprosy, tuberculosis, sore throat, liver disease, snake bite, as a pain reliever, and can also be used as sedative and carminative [12, 13]. Recently, stem and leaf methanol extracts of NF have been shown to have antidiabetic and analgesic effect [14]. Till date, no information on antioxidant activity and identification of phytochemicals from endosperm extract of ripe (EER) and unripe fruits (EEU) of NF has been documented. This work is essential, since it would determine the potency of the extract at different maturity levels. Hence, the objective of the present work is to evaluate the antioxidant capacity of EER and EEU of NF and also to identify its phenolic compounds.

## 2. Materials and Methods

### 2.1. Chemicals and Reagents

2-2′-Azino-bis (3-ethylbenz-thiazoline-6-sulfonic acid (ABTS), 2, 4, 6-tri (2-pyridyl)-l, 3, 5-triazine (TPTZ), aluminium chloride, tween-20, linoleic acid, chlorogenic acid, protocatechuic acid, kaempferol, rutin, hydroxybenzoic acid, beta carotene, quercetin, gallic acid, BHT, trichloroacetic acid, thiobarbituric acid, sodium azide, tris-HCl buffer, phosphate buffer, and Hepes were obtained from Sigma-Aldrich Co. (MO, USA). Folin-Ciocalteu reagent, trifluoroacetic acid (HPLC Grade), acetonitrile (HPLC Grade), and hydrogen peroxide were obtained from Merck (Darmstadt, Germany). All other chemicals and solvents used were of analytical grade.

### 2.2. Plant Material

**Figure 2:**
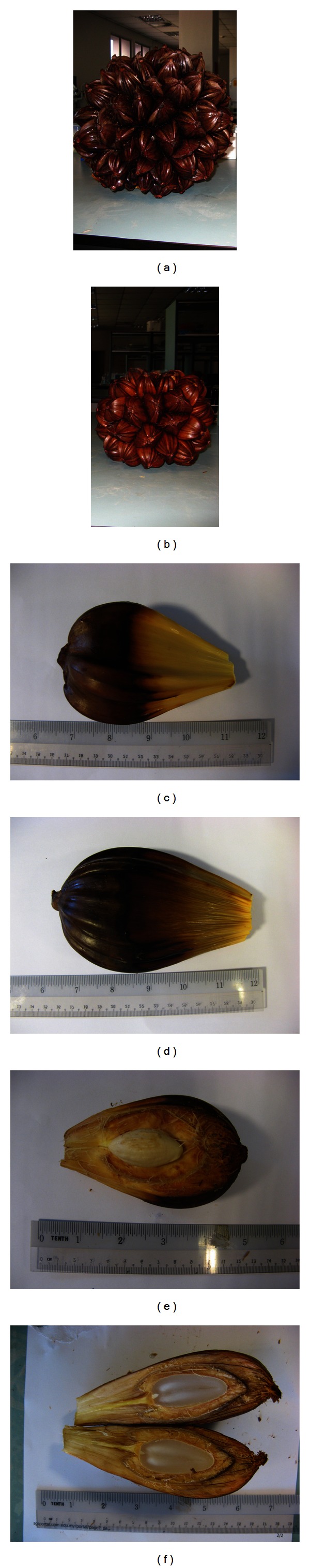
*Nypa fruticans *unripe (a) and ripe (b) whole fruit bunch, unripe (c) and ripe (d) individual fruit, and unripe (e) and ripe (f) endosperm.

Whole bunch of unripe (Figure 2(a)) and ripe fruits (Figure 2(b)) of *Nypa fruticans* at three and six month's maturity, respectively, were collected on the 6th of February 2011 from Kedah, Malaysia, with the assistance of Muda Agricultural Development Authority (MADA), Malaysia. Voucher specimens (NFUR622011 and NFR622011) are preserved at MADA office. The fruits were immediately transported to the laboratory of Universiti Putra Malaysia. Upon arrival, the fruits were washed under running tap water and air dried. The individual fruits (Figures 2(c) and 2(d)) were separated from the bunch and physical parameters of the fruits were measured (Table 1). Later, the fruits were manually separated to obtain the edible endosperm (Figures 2(e) and 2(f)). The endosperm (150 g) was kept in an oven maintained at 60°C for drying until constant weight was obtained. The dried portion was allowed to cool and be powdered and sieved (particle size of 20 mesh) to get uniform particle size. The dry powder was used for extraction.

### 2.3. Extraction

Dry powder (10 g) from unripe and ripe endosperms of NF was extracted separately with 50% ethanol (100 mL) using an orbital shaker (Unimax 1010, Heidolph, Germany). The shaker was maintained at a speed of 400 rpm and the extraction was carried out at 30°C for 1 h duration. The extracts were then filtered using Whatman filter paper (no. 4), concentrated, freeze dried using bench top freeze dryer (Virtis, NY, USA), and stored at −20°C until further analysis.

### 2.4. Determination of Total Phenolics Content

Total phenolics content (TPC) of the extracts was determined according to the method developed by Singleton and Rossi [15]. In brief, 100 *μ*L-aliquot of the extract was added to 2 mL of 20 g/L Na_2_CO_3_ solution. After 2 min, 100 *μ*L of 50% Folin-Ciocalteu reagent was added and the mixture was allowed to stand for 2 h at 25°C. The absorbance was measured at 750 nm using a spectrophotometer (UV 1601, Shimadzu Co., Ltd., Kyoto, Japan). The total phenolics content was determined using the standard gallic acid calibration curve and the results were expressed as milligram gallic acid equivalents per gram sample dry weight (mg GAE/g DW).

### 2.5. Determination of Total Flavonoid Content

Total flavonoid content (TFC) was measured using the aluminum chloride colorimetric assay described by Liu et al. [16]. An aliquot (0.1 mL) of the extract was mixed with 0.2 mL of 5% sodium nitrite. After 5 min, 0.2 mL of 10% aluminum chloride and 2 mL of 1 M sodium hydroxide were added and mixed vigorously. Absorbance was measured at 510 nm against a blank. The total flavonoid content was determined using a standard curve of rutin and the results were expressed as milligram rutin equivalent per gram sample dry weight (mg RE/g DW).

### 2.6. Analyses of Antioxidant Activities

#### 2.6.1. ABTS Radical Scavenging Activity

Radical scavenging activity of the samples against ABTS was carried out according to the method described by Re et al. [17]. ABTS radical cation was produced by reacting ABTS stock solution (7 mM) with 2.45 mM potassium persulfate and allowed the mixture to stand in the dark at room temperature for 16 h. The ABTS solution was diluted to obtain an absorbance of 0.70 at 734 nm and equilibrated at 30°C. ABTS solution (1 mL) was mixed with the extracts (100 *μ*L) at different concentrations (10, 50, 100, and 200 *μ*g/mL) and the decrease in absorbance after 6 min of incubation was monitored using spectrophotometer. Control received only ABTS solution, while distilled water was used as blank. The inhibition of ABTS radicals by the test samples was calculated as scavenging activity (%) = (Control optical density (OD) − sample OD/control OD)) × 100.

#### 2.6.2. DPPH Radical Scavenging Activity

DPPH radical scavenging activities were determined based on a method developed by Prasad et al. [6] with some minor changes. An aliquot of 0.1 mL of the extract at different concentrations (10, 50, 100, and 200 *μ*g/mL) mixed with 1 mL of DPPH (200 mM, dissolved in methanol). The reaction mixture was vortexed and incubated at 37°C in dark light for 30 min. The changes in absorbance were measured at 517 nm using a spectrophotometer. The inhibition of DPPH^*·*^ radicals was calculated as scavenging activity (%) = (Control OD − sample OD/control OD) × 100. BHT was used for comparison.

#### 2.6.3. Ferric Reducing Antioxidant Power (FRAP) Assay

Ferric reducing antioxidant power assay of the extracts was performed according to the method of Re et al. [17]. Working FRAP reagent was prepared by mixing acetate buffer (300 mM, pH 3.6) : 10 mM TPTZ solution in 40 mM HCL : 20 mM ferric chloride solution, in proportion of 10 : 1 : 1 (v/v/v). An aliquot (50 *μ*L) of appropriately diluted extract was mixed with 3 mL of freshly prepared FRAP reagent and mixed thoroughly. The reaction mixture was then incubated at 37°C for 30 min. Absorbance of the reaction mixture was read at 593 nm against a blank. The results were then calculated based on the calibration curve plotted using ferrous sulphate and expressed as mmol Fe^2+^/100 g dry weight.

#### 2.6.4. Beta Carotene Bleaching Assay

Beta carotene bleaching assay was performed according to the method of Velioglu et al. [18] with slight modifications. One milliliter of *β*-carotene solution (2 mg/mL dissolved in chloroform) was added into brown color round-bottom flask containing 0.02 mL of linoleic acid and 0.2 mL of Tween 20. The chloroform in the mixture was evaporated under vacuum and 100 mL of deionized water was added. The mixture was shaken vigorously to form an emulsion. The emulsion (1 mL) and 100 *μ*L of the extract at different concentrations (10, 50, 100, and 200 *μ*g/mL) were pipetted in different test tubes and incubated at 45°C for 2 h. Control received only the emulsion without any sample, while blank consists of emulsion without *β*-carotene and the extract. Absorbance of the solution was monitored at 470 nm. The rate of *β*-carotene bleaching was calculated as antioxidant activity coefficient (AAC) and calculated using the equation:

(1) $$\begin{array}{c} \text{AAC}=\left[{\text{A}}_{\text{s}\;(120)}-\frac{{\text{A}}_{\text{c}\;(120)}}{{\text{A}}_{\text{c}\;(0)}}-{\text{A}}_{\text{c}\;(120)}\right]\times 1000, \end{array}$$

where A_s (120)_ is absorbance of the sample at time 120 min, A_c (120)_ is absorbance of control at 120 min, A_c (0)_ is absorbance of control at 0 min, and A_c (120)_ is absorbance of control at 120 min. The higher the AAC values, the higher the antioxidant activity.

#### 2.6.5. Antioxidant Capacity by Phosphomolybdenum Method

Antioxidant capacity by phosphomolybdenum method was determined by the method of Prieto et al. [19]. An aliquot (0.1 mL) of the extracts at various concentrations (10, 50, 100, and 200 *μ*g/mL) was mixed with 1 mL of reagent solution (0.6 M sulphuric acid, 28 mM sodium phosphate, and 4 mM ammonium molybdate). The mixture was covered and incubated at 95°C for 90 min. After the mixture was cooled, it was centrifuged and absorbance of the supernatant was measured at 695 nm using a spectrophotometer. The antioxidant capacity was expressed as the absorbance value. A higher absorbance value indicates higher antioxidant capacity.

### 2.7. Identification and Quantification of Phenolic Compounds Using Ultra-High-Performance Liquid Chromatography (UHPLC)

Individual phenolic compounds in EEU and EER of NF were identified using validated UHPLC method described by Kong et al. [20] on an Agilent 1290 Infinity LC system (Agilent Technologies, Waldbronn, Germany). The system was equipped with binary pump, diode array detector, and an autosampler. A C-18 Zorbax Eclipse column (50 mm × 2.1 mm, I.D: 1.8 *μ*m, Agilent, Darmstadt, Germany) was used for polyphenol separation and it was maintained at 25°C. Five microliter of the sample was injected into the system and the elution program was set as follows. Mobile phase A is comprised of 0.1% trifluoroacetic acid (TFA) while mobile phase B contained acetonitrile, and the flow rate was set at 0.6 mL/min. The following linear gradient elution was carried out for separation of polyphenols, 15% B for 6 min; 25% B for 3 min; 60% B for 3 min; 80% B for 0.6; 100% B for 0.8 min. The total runtime was 14 min. UV-Vis absorption spectra were monitored by diode array detector (DAD) at 280 nm. The identification of phenolic compounds was achieved by comparison with retention times and UV-Vis absorption spectra with standards available. The phenolic compounds were quantified on the bases of their peak areas and calibration curves of the corresponding standards and then expressed as microgram per gram dry weight (*μ*g/g DW).

### 2.8. Statistical Analysis

Data were expressed as means ± standard deviations (SD) of three determinations and analyzed by SPSS V.13 (SPSS Inc., Chicago, USA). One way analysis of variance (ANOVA) and Duncan's multiple-range test were used to determine the differences among the means. *P* values of <0.05 were considered to be significantly different.

## 3. Results and Discussion

The fruit of NF was selected for the present investigation, since it is one of the important underutilized plants of Malaysia. Although the sap is often used as beverage [8], the fruits are discarded. Many underutilized fruits in Malaysia had been documented previously to be rich in antioxidants with many health benefits [5–7]. Weight (159.6 ± 7.7 g) and length (12.9 ± 0.7 cm) of ripe fruits were significantly higher (*P* < 0.05) than unripe ones (Table 1). Unripe endosperm is soft, juicy, and pale white in color (Figure 2(e)), while the ripe endosperm is hard and milky white in color (Figure 2(f)). These data are important for the food processing industry.

### 3.1. Total Phenolics and Flavonoid Contents

Phenolics and flavonoids present in fruits and vegetables have received considerable attention due to their potential antioxidant activities [21]. Phenolic compounds undergo a complex redox reaction with the phosphotungstic and phosphomolybdic acids present in Folin-Ciocalteu (FC) reagent. However, it should be also noted that some chemical groups of proteins, organic acids, and sugars present in the extracts might also react with FC reagent and therefore interfere with the result [22]. EEU of NF had significantly (*P* < 0.05) higher total phenolics content (135.6 ± 4.5 mg GAE/g), compared to EER (8.8 ± 2.0 mg GAE/g). In addition, higher total flavonoid content (*P* < 0.05) was also noticed in EEU (68.6 ± 3.1 mg RE/g), compared to EER (3.6 ± 0.4 mg RE/g).

### 3.2. Antioxidant Capacity Assay

Different antioxidant compounds could react through different mechanisms, and hence a single method alone cannot fully evaluate the antioxidant capacity of foods [23]. For this reason, different antioxidant capacity tests with different approaches and mechanisms were carried out in the present study.

#### 3.2.1. ABTS Assay

**Figure 3:**
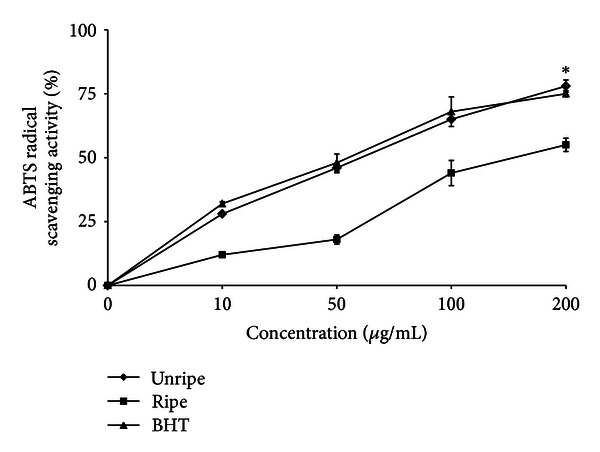
ABTS radical scavenging activity of unripe and ripe endosperm extracts of *Nypa fruticans*. *denotes significant differences at *P* < 0.05.

ABTS method is developed based on a decolorization technique. Once added, the antioxidant causes a reduction of ABTS, which could be measured at 734 nm. This assay is performed to measure the ability of antioxidants to inhibit radical cation induced by persulfate. Strong antioxidants have the ability to change the blue color of ABTS into light blue color. The change in absorbance is proportional to the antioxidant concentration [22].  Figure 3 illustrates the effect of the NF extracts against ABTS radicals. EEU exhibited significantly (*P* < 0.05) higher scavenging activity of 78 ± 1.2% at a concentration of 200 *μ*g/mL, higher than BHT standard (75 ± 2.6%). EER exhibited moderate activity of 55 ± 4.3%. The IC_50_ (concentration of the extract/sample to scavenge 50% ABTS radicals) values were also obtained. The lower the IC_50_ value the higher the antioxidant activity. The IC_50_ values of EEU (52 ± 2.7 *μ*g/mL) were parallel to BHT (51 ± 1.7 *μ*g/mL), but EER showed higher values (187 ± 4.7 *μ*g/mL). Our results are supported by similar findings by Gordon et al. [24], where unripe acacia fruits exhibited higher ABTS radical scavenging activity as compared to ripe fruits. Unripe cactus berry exhibited two times higher ABTS scavenging activity compared to ripe fruits [25]. The antioxidant activity of the extracts in the present investigation is probably due to the action of hydroxyl groups of phenolic compounds, which might act as hydrogen donors.

#### 3.2.2. DPPH Radical Scavenging Activity

**Figure 4:**
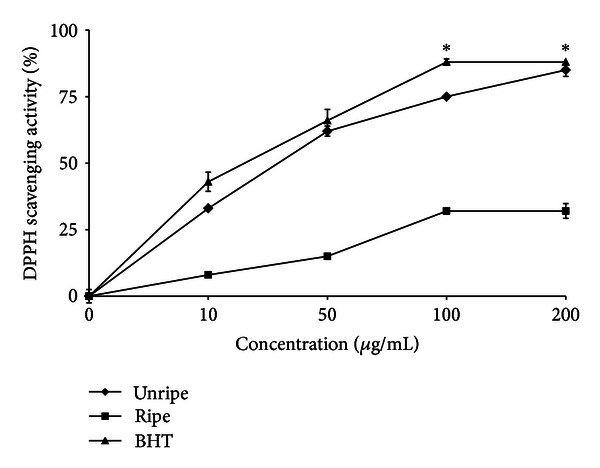
DPPH radical scavenging activity of unripe and ripe endosperm extracts of *Nypa fruticans*. *denotes significant differences at *P* < 0.05.

DPPH is a stable free radical and accepts an electron or hydrogen radical to become a stable diamagnetic molecule which is widely used to investigate radical scavenging activity. In DPPH radical scavenging assay, antioxidants react with DPPH and convert it to yellow coloured **α**,**α**-diphenyl-**β**-picryl hydrazine. The degree of discoloration indicates the radical-scavenging potential of the antioxidant [26]. DPPH radical scavenging activity of NF extracts and BHT increased as concentration increased (Figure 4). EEU exhibited the highest scavenging activity (85 ± 2.6%), significantly (*P* < 0.05) higher than EER (32 ± 2.8%) at a concentration of 200 *μ*g/mL, and was comparable to the scavenging activity of BHT (88 ± 1.2%). IC_50_ value of EEU (36 ± 1.2 *μ*g/mL) was more potent than EER (312 ± 4.8 *μ*g/mL). However, BHT showed the highest antioxidant activity with IC_50_ value of 12 ± 0.7 *μ*g/mL. It has been found that phenolics, flavonoids, and tocopherols scavenge DPPH radicals by their hydrogen-donating ability [22]. The results obtained in this investigation also reveal that the sample extracts act as free radical scavengers, which might be attributed to their electron-donating ability.

#### 3.2.3. Ferric Reducing Antioxidant Power (FRAP)

**Figure 5:**
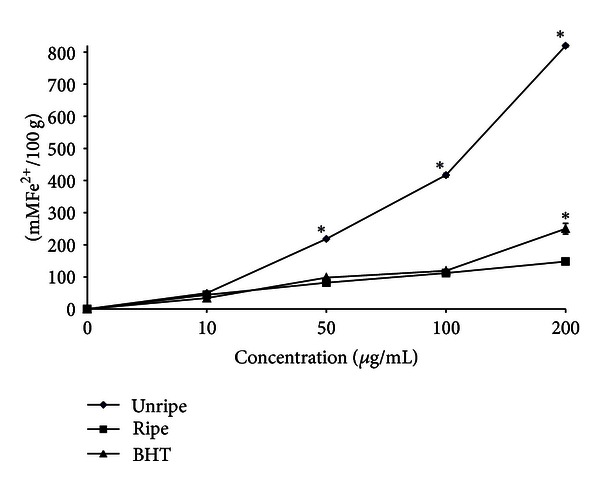
FRAP values of unripe and ripe endosperm extracts of Nypa fruticans. *denotes significant differences at *P* < 0.05.

Ferric reducing antioxidant power is widely used in evaluating antioxidant activity of plant polyphenols. Principally, FRAP assay treats the antioxidants in the sample as reductant in a redox-linked colorimetric reaction [22]. This assay is relatively simple and easy to conduct. FRAP assay measures the reducing potential of antioxidant to react on ferric tripyridyltriazine (Fe^3+^-TPTZ) complex and produce blue color of ferrous form which can be detected at absorbance of 593 nm [15]. Antioxidant compounds which act as reducing agent exert their effect by donating hydrogen atom to ferric complex and thus break the radical chain reaction [27]. In the present study, EEU exhibited the highest reducing power followed by BHT and EER (Figure 5). At 200 *μ*g/mL, the reducing power of EEU, BHT, and EER was 819 ± 4.3, 250 ± 16 and 147 ± 0.7 mmol Fe^2+^/100 g dry weight, respectively. The reducing power of fruit fractions is probably due to the action of hydroxyl group of the phenolic compounds which might act as electron donors.

#### 3.2.4. Beta Carotene Bleaching Assay

**Figure 6:**
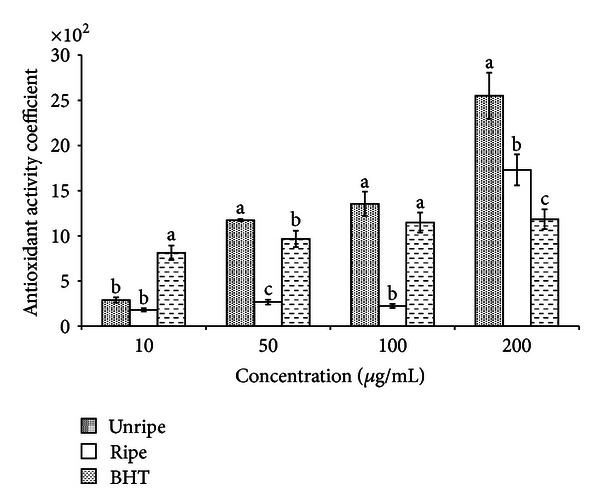
Antioxidant activity coefficient values determined by beta carotene bleaching method of unripe and ripe endosperm extracts of *Nypa fruticans.* For each treatment means in a row followed by different letters are significantly different at *P* < 0.05.

Beta carotene bleaching method is widely used to measure antioxidant activity of plant extracts. It is an *in vitro* assay that measures the inhibition of coupled autooxidation of linoleic acid and *β*-carotene. This method is based on lipid radicals as autooxidation products of linoleic acid which attack the double bonds of *β*-carotene, but in presence of antioxidants they can inhibit the oxidation and retain the yellowish-orange color of beta carotene and thus reduce their bleaching activity [28]. The bleaching activities of NF extracts were concentration dependent, and the activities increased as the concentration increased. EEU exhibited excellent antioxidant activity coefficient of 2550 ± 28 at a concentration of 200 *μ*g/mL, significantly higher (*P* < 0.05) than EER (1729 ± 19) and BHT (1183 ± 34) (Figure 6). The antioxidant activity of the extracts in the present investigation is probably due to the transfer of hydrogen atom from phenolic compounds to the free radical, therefore inhibiting bleaching of beta carotene.

#### 3.2.5. Antioxidant Capacity by Phosphomolybdenum Method

**Figure 7:**
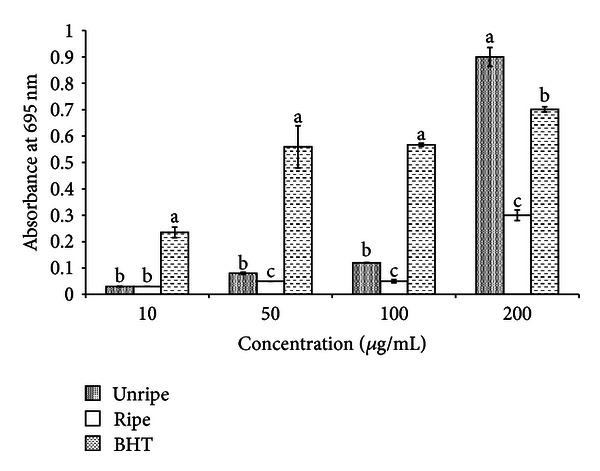
Antioxidant capacity of unripe and ripe endosperm extracts of *Nypa fruticans *determined by phosphomolybdenum method. For each treatment means in a row followed by different letters are significantly different at *P* < 0.05.

Antioxidant capacities of NF extracts were measured spectrophotometrically using phosphomolybdenum method, which is based on the reduction of Mo (IV) to Mo (V) by the sample analyte and subsequent formation of green phosphate/Mo (V) compounds with a maximum absorption at 695 nm [19]. A high absorbance value of the sample indicates its strong antioxidant activity.  Figure 7 shows the total antioxidant capacities of NF extracts and BHT. All the extracts showed a concentration-dependent activity. The total antioxidant activity of EEU at 200 *μ*g/mL was 0.9 (Figure 3), significantly higher (*P* < 0.05) than EEU (0.3) and BHT (0.7). However, the total antioxidant activity of BHT at all other concentrations tested was higher than other extracts. Previously, Jayaprakasha et al. [29] indicated that total antioxidant activity of citrus was due to the presence of bioactive compounds in the form of phenolics and flavonoids. Hence, probably in the present investigation, the antioxidant capacity might be attributed to the reducing activity of phenolic compounds.

### 3.3. Identification and Quantification of Phenolic Compounds

**Figure 8:**
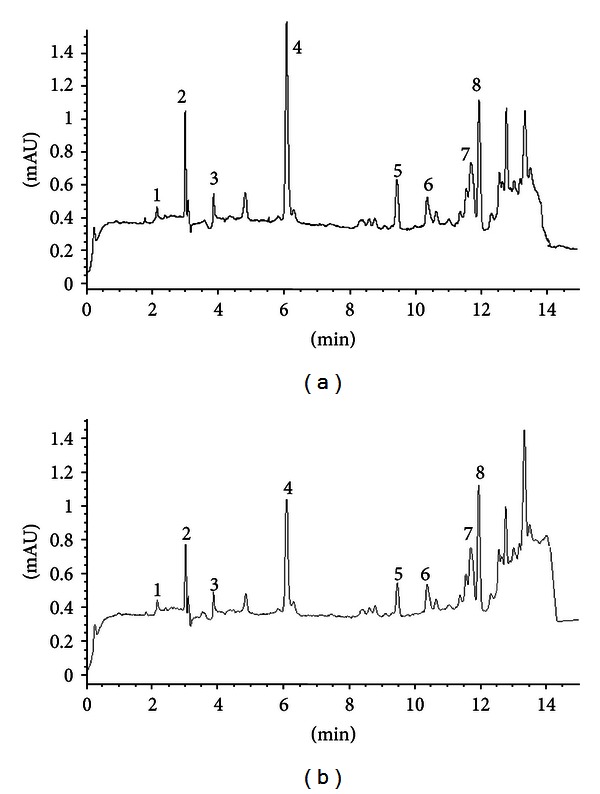
HPLC chromatogram of unripe (a) and ripe endosperm extract (b) of *Nypa frutican*. The peak identifications are (1) gallic acid, (2) protocatechuic acid, (3) hydroxybenzoic acid, (4) chlorogenic acid, (5) rutin, (6) cinnamic acid, (7) quercetin, and (8) kaempferol.

HPLC chromatograms from EEU (Figure 8(a)) and EER (Figure 8(b)) of NF are provided. Eight phenolic compounds were identified where chlorogenic acid, protocatechuic acid, and kaempferol were the major compounds. Among all the phenolic compounds identified, chlorogenic acid was the highest in EEU (14.5 ± 1.30 *μ*g/g) and EER (9.7 ± 1.10 *μ*g/g), while the lowest was gallic acid (Table 2). Protocatechuic acid occupied the second major compound as noted in EEU (5.5 ± 0.40 *μ*g/g) and EER (3.8 ± 0.50 *μ*g/g), followed by kaempferol. In addition, hydroxy benzoic acid, rutin, cinnamic acid, and quercetin were detected in minor amount. Phenolic compounds (chlorogenic acid, protocatechuic acid, rutin, and hydroxybenzoic acid) of EEU were significantly higher (*P* < 0.05) than EER.

Our results are in good agreement with Monde et al. [30], where chlorogenic acid was reported as the major compound in oil palm fruits. In addition caffeic acid, rutin and quercetin were also detected. Da Silva Campelo Borges et al. [31] have identified gallic acid, hydroxy benzoic acid, ferulic acid, and quercetin from jussara palm plant. Gordon et al. [24] reported from acacia palm that protocatechuic acid, chlorogenic acid, hydroxy benzoic acid and gallic acid decreased as fruit maturity increased. In support to current findings, Herrera-Hernández et al. [25] reported high gallic acid, ellagic acid, caffeic acid, and quercetin in unripe fruits, when compared to ripe fruits.

Antioxidant activity and phenolic content of EEU determined in the present study were higher compared to EER. This is in good agreement with other researchers, where they had reported parallel results [25, 32, 33]. Phenolic compounds are synthesized rapidly during the early stages of fruit maturity. Once the fruit matures, decrease in phenolic concentration is noticed due to the dilution caused by cell growth [34]. Reduction of primary metabolism in the ripe fruit due to lack of substrates necessary for the biosynthesis of phenolic compounds also results in decrease of phenolics compounds. In addition, polymerisation, oxidation, and conjugation of bound phenolics during maturation could also result in decrease of phenolic composition [32].

The total phenolics content of NF is higher when compared to acai palm (123.1 GAE/g) [24] and date palm (4.8 GAE/g) [35]. Statistical correlations between total phenolics content and antioxidant activities were also determined. Total phenolics content exhibited the highest associations with FRAP (*r* = 0.9815), total antioxidant capacity (*r* = 0.9523), and DPPH (*r* = 0.8594). For total flavonoid content, its correlation with FRAP and antioxidant capacity was low (*r* = 0.249 and 0.315 resp.), while negative correlation was observed for other antioxidant assays. Thus, the amount of phenolic compounds could be used as an important indicator of antioxidant capacity. This also clearly indicated that total phenolics in EEU and EER of NF are major contributors for antioxidant activities since they have a high correlation, while flavonoids are not the major contributors for antioxidant activities. In addition, antioxidant activity could also be contributed by synergistic action of other phytochemicals which are not determined in the current study such as carotenoids and polysaccharides. Previously, many authors have reported a positive correlation between total phenolics content and antioxidant actity [16, 34]. Liu et al. [16] reported negative correlation between antioxidant activity and total flavonoid content.

## 4. Conclusion

Unripe endosperm extract of *Nypa fruticans* showed high total phenolics, total flavonoid content, and antioxidant capacities as compared to ripe endosperm extract. Chlorogenic acid, protocatechuic acid, and kaempferol were identified as major compounds in the extract. Thus, unripe endosperm extract of NF could be used as natural antioxidant. Further investigations on the evaluation of nutritional composition and other therapeutic uses of *Nypa fruticans* endosperm extracts are worth investigating.

## Figures and Tables

**Table 1:** Physical parameters of *Nypa fruticans *fruit.

	Unripe	Ripe
Whole fruit bunch		
Weight (kg)	7.2 ± 0.19^b^	16.1 ± 0.16^a^
Length (cm)	25.5 ± 2.5^b^	34.5 ± 2.7^a^
Perimeter (cm)	81.2 ± 4.5^b^	108.9 ± 6.3^a^
Individual fruit		
Weight (g)	138.1 ± 5^b^	159.6 ± 7.7^a^
Length (cm)	11.1 ± 0.5^b^	12.9 ± 0.7^a^
Breadth (cm)	7.8 ± 0.5	8.1 ± 0.3
Endosperm		
Weight (g)	3.6 ± 1.5^b^	19.6 ± 0.8^a^
Length (cm)	3.0 ± 0.2^b^	4.5 ± 0.5^a^
Perimeter (cm)	5.8 ± 0.7^b^	10.1 ± 0.5^a^

For each treatment means in a row followed by different letters are significantly different at *P* < 0.05.

**Table 2:** Phenolic compound composition of unripe and ripe endosperm extracts of *Nypa fruticans* (*μ*g/g DW of plant material).

Peak number	Compound	Retention time (min)	*λ* max (nm)	Unripe	Ripe
1	Gallic acid	2.1	272	0.47 ± 0.02	0.46 ± 0.01
2	Protocatechuic acid	2.9	294	5.52 ± 0.40^a^	3.88 ± 0.50^b^
3	Hydroxybenzoic acid	3.9	256	1.10 ± 0.07^a^	0.87 ± 0.06^b^
4	Chlorogenic acid	6.1	326	14.5 ± 1.30^a^	9.7 ± 1.10^b^
5	Rutin	9.5	354	2.7 ± 0.04^a^	2.2 ± 0.07^b^
6	Cinnamic acid	10.7	272	1.1 ± 0.01	0.9 ± 0.02
7	Quercetin	11.6	372	1.73 ± 0.02	1.74 ± 0.7
8	Kaempferol	11.9	364	3.2 ± 0.40	3.0 ± 0.10

For each treatment means in a row followed by different letters are significantly different at *P* < 0.05.
